# Rats and Mice as Enemies of Mankind
**Rats and Mice as Enemies of Mankind.* By M. A. C. Hinton. (London: The British Museum (Natural History), Cromwell Road, S. W. 7. Price 1s.)


**Published:** 1919-03-22

**Authors:** 


### RATS AND MICE AS ENEMIES OF MANKIND.*

This pamphlet—really a neat little volume of some seventy pages—follows as No. 8 in the Economic Series, dealing with the many pests which invade the homes of men, and proves to be a very valuable addition to the set. The author's account shows how urgent is the necessity for co-ordinated action in reducing the numbers of these small animals taking such enormous toll of our food and stores and playing so important a part in the dispersal of several of the most severe diseases affecting the human race.

A detailed and highly interesting description of the British representatives of the family *Muridæ*, with their habits, origin, migrations, and breeding, is followed by a concise demonstration of the many ways in which they menace the public welfare. These fall naturally into two main classes, the economic and the medical. By burrowing everywhere, gnawing through partitions and joists, damaging even gas and water pipes, carrying matches into their combustible nests and igniting them, and stripping the insulation from electric wires, the rodents are often responsible for serious structural collapse and damage to property. In case of transit by rail or sea large quantities of all varieties of merchandise are annually destroyed or more or less ruined; while in houses, hospitals, &c., a most catholic taste is displayed—food, furniture, linen, books, pets, and sometimes even man (alive or dead) being indiscriminately attacked. An enormous toll is levied upon farmers, millers, grain merchants, and consumers; in fact, people who keep live-stock, for profit or pleasure, often feed regularly, if unwittingly, some hundreds of rats and mice as well. The historical and statistical details of these ravages are most interesting, and one stands amazed at the magnitude of the authorities' estimates. In 1909 the capital employed in this country to produce rat exterminating apparatus had grown to £2,000,000, while the annual damage caused, omitting the destruction caused by rats in our ships and relating solely to the rural and urban activities of the animals, actually amounted to £15,000,000!

But this loss becomes insignificant when the risk of disease conveyed by these pests is realised. The financial side directly affects only a limited number of people, and many of these ignorant of its relative extent, but the menace to health is real enough to affect every member of the community. The list of diseases disseminated by rodents will undoubtedly increase with advancing biological and medical studies, but, while explaining the direction of this probability, the pamphlet discusses, in detail, only the better known. Briefly, but very carefully, are put forward bubonic and pneumonic plague, trichinosis, sokodu or rat-bite fever, influenza, foot-and-mouth disease, and dysentery. And, finally, with regard to the stores and kitchens of our houses and restaurants, to use the author's own words, "into these places, besides contaminating our food with its own germ-laden dejecta and parasites, the animal brings a wealth of indescribable filth from its favourite haunts in the adjoining sewers and drains. At this point one may leave the reader to his own reflections."

Preventative measures are set forth under the following headings: Protection of food supplies, removal and destruction of refuse, rat-proofing of buildings, protection of drains, fumigation of ships, and the clearing of quays and railway depôts. In this connection comes the question of trapping, poisoning, and the use of virus. The description of the relative values of these methods is most interesting.

"If a general campaign against rats should be organised throughout Britain, it should proceed on some such lines as the following: The country should be divided into districts, each having as far as possible water for its boundaries. Work in each district should commence at the boundaries and proceed gradually towards the centre; the more the work is supplemented by individual effort the more effectual it will be; and the full co-operation of all landowners and farmers will be essential. Systematic operations should commence immediately after the harvest, and they should be continued into the spring, the ground being traversed more than once if possible. Trapping, of course, should be done throughout the year. . . . It is suggested that rat-catching offers a good field of employment for many disabled soldiers and sailors."

The urgency of the situation is fully demonstrated, and this small publication should be appreciated by all who take close interest in the welfare of the public from either a medical or economic point of view. Advantage may well be taken of means and methods suggested by an author, himself so obviously an authority.

*Rats and Mice as Enemies of Mankind. By M. A. C. Hinton. (London: The British Museum (Natural History), Cromwell Road, S.W. 7. Price 1s.)

